# Clinical translation of stem cell therapy for spinal cord injury still premature: results from a single-arm meta-analysis based on 62 clinical trials

**DOI:** 10.1186/s12916-022-02482-2

**Published:** 2022-09-05

**Authors:** Zhizhong Shang, Mingchuan Wang, Baolin Zhang, Xin Wang, Pingping Wanyan

**Affiliations:** 1 The First Clinical Medical College of Lanzhou University, Lanzhou, 730000 China; 2 Chengren Institute of Traditional Chinese Medicine, Lanzhou, 730000 Gansu Province China; 3 Department of Spine, Changzheng Hospital, Naval Medical University, Shanghai, 200003 China; 4 Gansu University of Chinese Medicine, Lanzhou, 730000 China; 5 The Second Hospital of Lanzhou University, Lanzhou, 730000 China

**Keywords:** Stem cells, Spinal cord injury, Meta-analysis, Clinical trials

## Abstract

**Background:**

How much scientific evidence is there to show that stem cell therapy is sufficient in preclinical and clinical studies of spinal cord injury before it is translated into clinical practice? This is a complicated problem. A single, small-sample clinical trial is difficult to answer, and accurate insights into this question can only be given by systematically evaluating all the existing evidence.

**Methods:**

The PubMed, Ovid-Embase, Web of Science, and Cochrane databases were searched from inception to February 10, 2022. Two independent reviewers performed the literature search, identified and screened the studies, and performed a quality assessment and data extraction.

**Results:**

In total, 62 studies involving 2439 patients were included in the analysis. Of these, 42 were single-arm studies, and 20 were controlled studies. The meta-analysis showed that stem cells improved the ASIA impairment scale score by at least one grade in 48.9% [40.8%, 56.9%] of patients with spinal cord injury. Moreover, the rate of improvement in urinary and gastrointestinal system function was 42.1% [27.6%, 57.2%] and 52.0% [23.6%, 79.8%], respectively. However, 28 types of adverse effects were observed to occur due to stem cells and transplantation procedures. Of these, neuropathic pain, abnormal feeling, muscle spasms, vomiting, and urinary tract infection were the most common, with an incidence of > 20%. While no serious adverse effects such as tumorigenesis were reported, this could be due to the insufficient follow-up period.

**Conclusions:**

Overall, the results demonstrated that although the efficacy of stem cell therapy is encouraging, the subsequent adverse effects remain concerning. In addition, the clinical trials had problems such as small sample sizes, poor design, and lack of prospective registration, control, and blinding. Therefore, the current evidence is not sufficiently strong to support the clinical translation of stem cell therapy for spinal cord injury, and several problems remain. Additional well-designed animal experiments and high-quality clinical studies are warranted to address these issues.

**Supplementary Information:**

The online version contains supplementary material available at 10.1186/s12916-022-02482-2.

## Background

According to the Global Burden of Diseases report, the age-standardized rate of spinal cord injury (SCI) is 13 (11 to 16) per 100,000 people, accounting for 27,042,505 cases (24,976,608 to 30,148,230) worldwide. In 2016 alone, there were 935,000 new cases of SCI [1]. A variety of treatment strategies, such as drugs (methylprednisolone, ganglioside), surgery (decompression and fusion surgery), and rehabilitation therapy (electrical stimulation, functional exercise), have been used widely in the clinic [2]. However, their efficacy is limited due to severe pathophysiological injuries after SCI, such as neuronal necrosis and apoptosis, ischemia-reperfusion injury, and the demyelination and degeneration of spinal axons. Currently, less than 1% of patients with SCI show complete recovery of neurological function [3, 4]. Therefore, new and more effective treatments are urgently required.

Decades of research have deepened our understanding of the mechanisms underlying injury, neural repair, and regeneration in SCI [5, 6]. Studies have shown that stem cells can protect and regenerate the injured spinal cord through neuroprotection, immunomodulation, axon sprouting and/or regeneration, neuronal relay formation, and myelin regeneration, among other mechanisms [4, 7]. Therefore, stem cell therapy has attracted great attention as a treatment strategy for SCI.

Stem cell therapy has shown great progress in preclinical research. Moreover, it has shown high therapeutic efficacy in other conditions, such as hematological malignancies, burns, and corneal transplants. Thus, it has attracted the attention of countless eager patients, clinicians, pharmaceutical companies, and the media [8–10]. Several interested parties are eagerly calling for the clinical translation of stem cell therapy for SCI, leading to a large number of clinical trials for stem cells. To accelerate this process, findings from rodent models were directly validated in human patients instead of using a step-wise testing approach in rodent models, followed by canine and feline models, primate models, and finally humans. As a result, a vast majority of preclinical trials have been conducted in rodents, and there are fewer than 20 studies in large animal models, accounting for < 2% of all preclinical research [11]. On the contrary, the number of related clinical trials has exceeded 100. Subsequently, unproven stem cell therapies in which patients receive autologous transplants could be performed without sufficient evidence, supervision, or informed consent [12, 13].

More notably, owing to the explosive growth of basic and clinical research in the field of stem cell therapy, other important issues may be neglected in the interest of rapidly advancing the clinical translation of stem cell therapy. Of these, the first and most important issue is the safety of stem cells. Stem cell therapy can lead to tumor formation, inappropriate migration, secondary injury, infection, and other adverse effects (AEs) [14]. The second is the ethical concerns related to the source, extraction, and transformation of stem cells. For example, human embryonic stem cells (hESCs) are known to show effective outcomes. However, are these embryos a potential form of life or just cells for research? What are the legal implications of destroying embryos? Should hESCs be derived from excess gametes or blastocysts during in vitro fertilization, aborted fetuses, voluntarily donated germ cells, or embryos produced for research purposes? In addition, although umbilical cord mesenchymal stem cells (UCMSCs) can be derived from discarded neonatal umbilical cords, they are the private property of mothers and neonates and are genetic resources protected by the law. These cells may cause allergy, hemolysis, and chronic toxicity after transplantation. Furthermore, they may carry pathogens and genetic mutations. Induced pluripotent stem cells (iPSCs) have the ability of unlimited proliferation and multi-directional differentiation. However, undifferentiated iPSCs can develop teratomas due to gene insertion mutations and uncontrolled proliferation and differentiation, leading to ethics-related debates [15, 16]. Currently, one of the most pressing ethical issues is the growing number of medical institutions that offer unproven stem cell-based treatments [17]. Finally, there are regulatory controversies over stem cells, and whether these regulations should be strict or lenient is often debated. The US Food and Drug Administration (FDA) has established a strict three-level regulatory system of “regulations-regulation-guiding principles” that requires research on stem cells to be subject to FDA review. Recently, the FDA has further stepped up its regulatory and enforcement activity for stem cell therapy to ban clinics and companies that could undermine the health of the entire regenerative medicine industry. In contrast, stem cell regulation in Japan is relatively less strict. The regulation of stem cells in Japan is subject to different levels of review. There are three risk levels based on the source of cells, processing methods, and the scope of application. With regulatory reforms, stem cell products could receive conditional approval after positive clinical findings in only 10 patients [18]. Benefiting from its comprehensive legal system and special approval policies, Japan has become a global leader in technological research and the development of cell therapy products. In other countries, such as China and India, several problems surrounding the regulation of stem cells remain. The regulatory boards only propose the basic principles for the development and evaluation of cell therapy products, and there is no implementation of stratified supervision based on the characteristics and applications of stem cells [19]. In addition, the necessary issues for the successful clinical application of stem cells remain unaddressed, including the risk-benefit ratio, mode of transplant strategy, oversights, conflicts of interest, surgical innovation, informed consent, and patient vulnerability [13].

Undoubtedly, despite several challenges, the initial success of stem cells in animal experiments and patients is indeed a cause for celebration and inspiration. It has brought immense hope of recovery for countless patients with SCI. However, experts must remain aware that there is still a lot of work to be done. Although systematic reviews of stem cell therapy for SCI have been published previously, they have had certain problems and limitations [20, 21]. Most of the current studies are single-arm, early-stage clinical trials with the main purpose of evaluating the safety of stem cells. However, published systematic reviews have only included randomized controlled trials, focusing on the effectiveness of stem cells, resulting in a limited number of included studies. Therefore, such systematic reviews do not fully analyze the existing data. Furthermore, they do not address the most important aspect, i.e., the safety of stem cell therapy and the feasibility of its translation from laboratory research to clinical practice. As such, it is unclear if we now have enough evidence to support the immediate clinical translation of stem cell therapy. Therefore, we performed a single-arm meta-analysis that included all the published clinical data and did not exclude studies based on the type of clinical research. Accordingly, we systematically evaluated the benefits and risks of stem cell therapy in patients with SCI in order to examine the feasibility of its clinical translation.

## Methods

This systematic review and meta-analysis followed the Preferred Reporting Items for Systematic Reviews and Meta-Analyses (PRISMA) guidelines [22]. Although the study was not registered with the PROSPERO database or other comparable databases, we verified that no similar study has been registered before undertaking this study.

### Inclusion and exclusion criteria

#### Patients and diseases (P)

Patients with SCI.

#### Interventions (I)

Stem cells, no source restrictions.

#### Control (C)

There was no restriction based on whether the study had a control group.

#### Outcome (O)

(1) Primary outcome measure (safety indicator): AEs involving the nervous system, musculoskeletal system, digestive system, cardiovascular system, and other systems; (2) secondary outcome measures (effectiveness indicator): American Spinal Cord Injury Association Impairment Scale (ASIA) score improvements of at least one grade and improvements in the urinary system and gastrointestinal function.

#### Type of study (S)

Descriptive research, analytical research, and experimental research.

#### Exclusion criteria

The following are the exclusion criteria:

(1) Repeatedly published research, (2) unavailablity of the full text, (3) sample size of less than 3 subjects, and (4) inclusion of patients with other serious disorders.

### Data sources and searches

Candidate studies were identified through searches of the PubMed, Web of Science, Cochrane, and Embase databases from inception until February 10, 2022. The following terms were combined to generate search keywords: (Spinal cord injury OR Spinal injury OR Spinal Cord Trauma OR Spinal Cord Transection OR Spinal Cord Laceration OR Post-Traumatic Myelopathy OR Spinal Cord Contusion) AND (stem cell OR stem cells). Further details of the search strategy are shown in Additional file 1: Table S1. We also reviewed studies included in previous systematic reviews and the reference lists of the included papers to identify other relevant studies.

### Literature screening and data extraction

Two trained researchers selected the papers and stringently extracted the data based on the inclusion/exclusion criteria independently, and the selections were cross-checked. Disagreements were resolved by a third researcher through a common consensus. Data were extracted according to the pre-established full-text data extraction checklist, which included (1) basic characteristics of studies such as authors, year of publication, country, and type of study; (2) patient characteristics such as gender, age, sample size, and location and duration of injury; (3) types and sources of stem cells and the dose and route of transplantation; (4) key elements of bias risk assessment; and (5) outcome measures, i.e., effectiveness and safety indicators.

### Methodological quality assessment

The methodological index for non-randomized studies (MINORS) was used to evaluate the methodological quality of the included studies. The following eight items were evaluated: (1) a clearly stated aim, (2) inclusion of consecutive patients, (3) prospective collection of data, (4) endpoints appropriate to the aim of the study, (5) unbiased assessment of the study endpoint, (6) follow-up period appropriate to the aim of the study, (7) loss to follow-up less than 5%, and (8) prospective calculation of the study size. Each item was scored on a scale of 0–2, where 0 indicated unreported, 1 indicated underreported, and 2 indicated adequately reported.

### Statistical analysis

STATA 16.0 (Stata Corp, College Station, TX) was used for the meta-analysis of the incidence of each outcome event. Heterogeneity among studies was estimated using the *χ*^2^ test and the *I*^2^ statistics. If *p* was < 0.1 and/or *I*^2^ was > 50%, there was considered to be heterogeneity among the included studies, and the random-effects model was used for combined analysis. Otherwise, the fixed-effects model was used. In addition, in order to explore the safety and efficacy of stem cells from different sources, an additional subgroup analysis was performed.

## Results

### Literature search

**Fig. 1:**
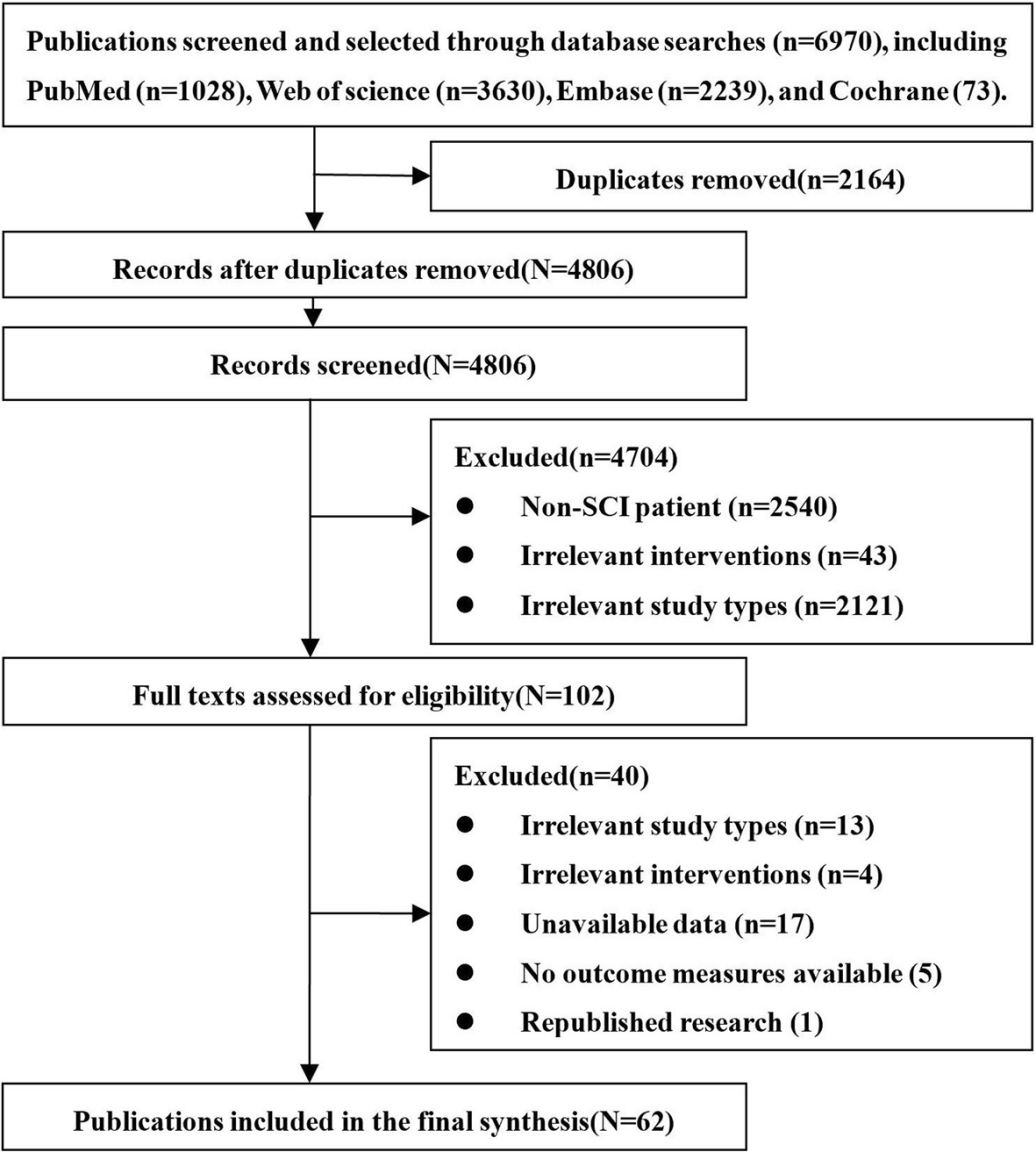
Flow diagram for study inclusion

A total of 6970 related studies were obtained from the preliminary search. After removing duplicate papers, a total of 4806 unique studies remained. Based on a preliminary screening of the titles and abstracts, we excluded 2540 non-clinical studies (animal studies, cell research, and in vitro experiments, etc.), 43 studies on non-stem cell interventions (exosome derived from stem cells, etc.), and 2122 articles that were not research papers (reviews, conference abstracts, letters to editors, editorials, etc.). The full text of the remaining 102 papers was screened. Some studies were further excluded due to the non-compliance of research type, non-compliance of intervention, lack of access to data, unreported outcome indicators, and duplication. Finally, 62 studies were included. The article screening process is shown in Fig. 1.

### Basic information of included studies

The 62 studies included 42 single-arm studies and 20 controlled studies. A total of 2439 patients were included, and the sample size of each study ranged from 4 to 297. The age of the patients was between 6 and 65 years. The sites of SCI included the cervical, thoracic, and lumbar spinal cord, and the injury duration ranged from 3 days to 341 months. The types of stem cells included bone marrow mesenchymal stem cells (BMSCs, 38 studies), adipose tissue-derived mesenchymal stem cells (ADMSCs, 2 studies), UCMSCs (9 studies), cord blood stem cells (CBSCs, 1 study), ESCs (3 studies), hematopoietic stem cells (HSCs, 5 studies), and neural stem cells (NSCs, 5 studies). The number of transplanted stem cells varied greatly, ranging from 5 × 10^4^ to 1.98 × 10^10^. The transplantation routes included intrathecal, scaffold-loaded, intralesional, venous, arterial, and subdural administration. The follow-up period ranged from 6 to 60 months. The basic information of the included studies is shown in Additional file 1: Table S2 [23–84].

### Methodological quality assessment

**Fig. 2:**
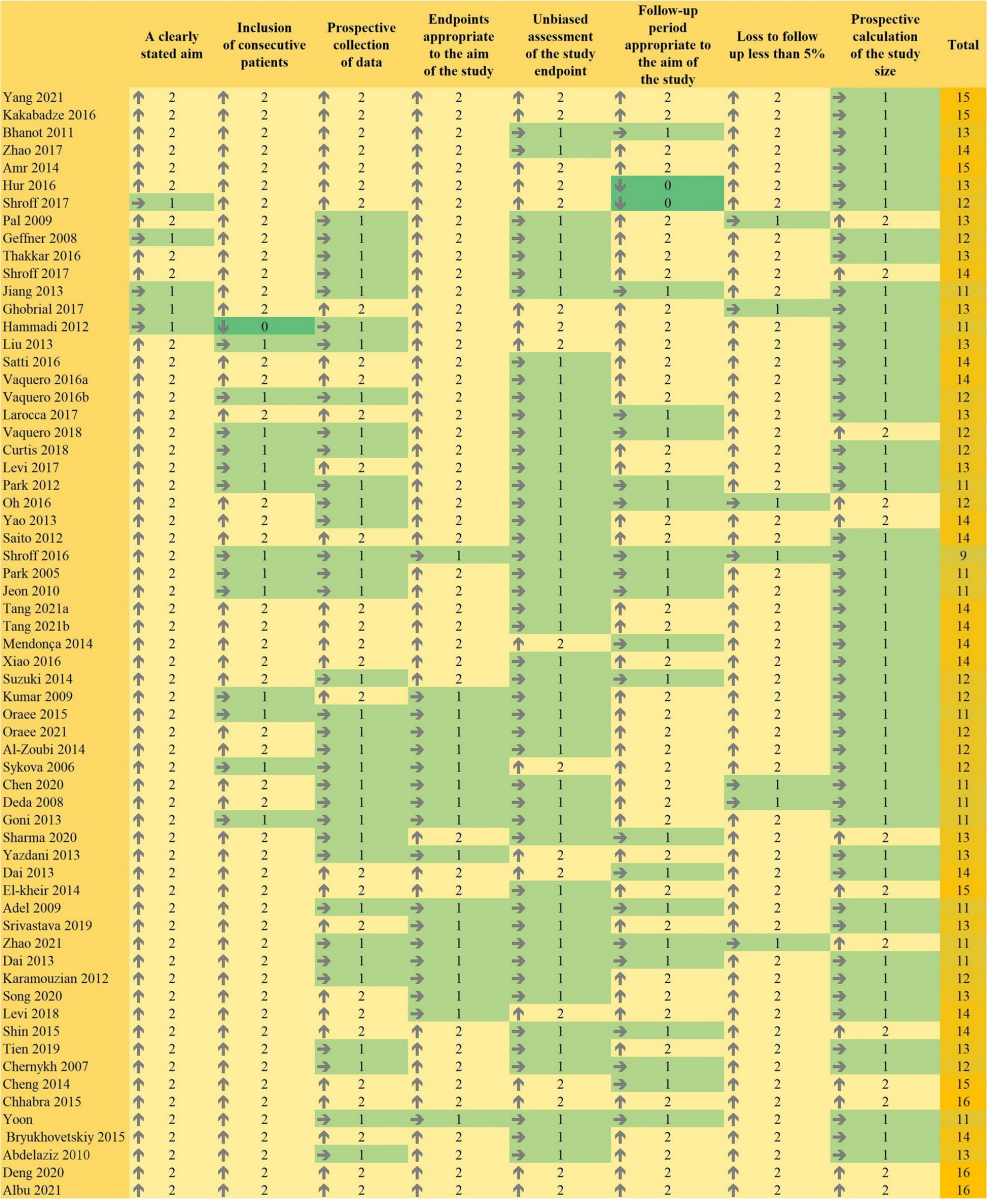
Methodological quality assessment results (0, high risk; 1, medium risk; 2, low risk)

Of the 62 studies, 57 clearly stated the purpose of the study, 48 clearly reported the criteria for the inclusion and exclusion of patients, and the endpoint indicators of 44 studies appropriately reflected the purpose of the study. However, only 28 studies developed a research protocol before the trial, and only 17 evaluated the results in a blinded manner. In addition, 22 studies had a short follow-up period (12 months), 7 studies had a high rate of loss to follow-up (> 5%), and 49 studies did not fully calculate and statistically analyze the sample size, outcome indicators, and other data. The results of the quality evaluation are shown in Fig. 2.

### Meta-analysis results

#### Primary outcome measures

A total of 41 studies reported at least one AE. Given the large heterogeneity among the included studies, we used the random effects model for meta-analysis.

We classified the AEs based on the organ system involved and thus analyzed the AEs in each system. (1) AEs of the nervous system: This group included 11 types of AEs, with a total incidence rate of 14.9% [11.3%, 18.9%]. Among them, the incidence of neuropathic pain was the highest at 25.2% [14.2%, 37.8%]. (2) AEs of the musculoskeletal system: This group included 6 kinds of AEs, with a total incidence rate of 20.4% [9.3%, 33.9%]. Among them, the incidence of muscle spasms was the highest at 25.5% [13.2%, 39.8%]. (3) AEs of the digestive system: This group included 3 kinds of AEs, with a total incidence rate of 11.5% [3.5%, 22.5%]. Among them, the incidence of vomiting was the highest at 21.6% [8.9%, 37.2%]. (4) AEs of the cardiovascular system: This group included 4 kinds of AEs, with a total incidence rate of 7.6% [3.7%, 12.6%]. Among them, the incidence of hypertension was the highest at 12.7% [8.7%, 17.3%]. (5) Other AEs: This group included pressure ulcers, itching and rash, lung infection, and urinary tract infection. Details are provided in Table 1.

**Table 1:** Meta-analysis results of AEs

AEs	Stem cell types	Number of studies	Incidence	Heterogeneity (***I***^**2**^)
**AEs of the nervous system**				
Neuropathic pain	Total	20	25.2% [14.2%, 37.8%]	84.514%
	BMSCs	17	27.9% [15.1, 42.5]	86.636%
	UCMSCs	2	14.0 [2.6, 30.5]	0.0%
	NSCs	1	8.3 [0.2, 38.5]	/
Abnormal feeling	Total	4	24.8% [10.5%, 41.8%]	0.0%
	BMSCs	4	24.8% [10.5%, 41.8%]	0.0%
Fever	Total	18	15.5% [10.0%, 21.8%]	80.528%
	BMSCs	9	18.6% [7.1%, 33.2%]	83.669%
	UCMSCs	5	11.6% [8.4%, 15.1%]	0.0%
	HSCs	3	8.9% [0%, 26.8%]	94.602
	ESCs	1	13.2% [8.9%, 18.7%]	/
	CBSCs	1	12.0% [2.5%, 31.2%]	/
Headache	Total	19	13.5% [8.4%, 19.5%]	80.332%
	BMSCs	10	16.2% [8.1%, 25.9%]	70.036%
	UCMSCs	4	5.5% [0.6%, 13.4%]	44.522%
	NSCs	2	19.2% [7.9%, 33.4%]	0.0%
	ADMSCs	1	21.4% [4.7%, 50.8%]	/
	ESCs	1	11.3% [7.3%, 16.4%]	/
	HSCs	1	12.4% [8.2%, 17.7%]	/
Abnormal autonomic reflexes	Total	1	10.3% [2.2%, 27.4%]	/
	NSCs	1	10.3% [2.2%, 27.4%]	/
Cerebrospinal fluid leak	Total	3	7.7% [1.7%, 16.3%]	0.0%
	BMSCs	2	5.8% [0%, 17.4%]	0.0%
	NSCs	1	10.3% [2.2%, 27.4%]	/
Meningismus	Total	1	3.5% [1.4%, 7.0%]	/
	HSCs	1	3.5% [1.4%, 7.0%]	/
Pseudomeningocele	Total	1	3.4% [0.1%, 17.8%]	/
	NSCs	1	3.4% [0.1%, 17.8%]	/
Lethargy	Total	1	2.0% [0.5%, 5.0%]	/
	HSCs	1	2.0% [0.5%, 5.0%]	/
Dizziness	Total	2	1.9% [0.9%, 3.2%]	57.3%
	UCMSCs	1	1.3% [0.4%, 3.0%]	/
	HSCs	1	3.5% [1.4%, 7.0%]	/
Mental disorder	Total	2	1.0% [0%, 3.2%]	77.7%
	UCMSCs	1	20.0% [5.7%, 43.7%]	/
	HSCs	1	1.0% [0.1%, 3.5%]	/
**AEs of the musculoskeletal system**				
Muscle spasms	Total	11	25.5% [13.2%, 39.8%]	90.162%
	BMSCs	8	24.1% [10.5%, 40.6%]	84.328%
	UCMSCs	1	24.1% [10.3%, 43.5%]	/
	NSCs	1	5.3% [0.1%, 26.0%]	/
	HSCs	1	54.5% [47.3, 61.5%]	/
Increased muscle tone	Total	4	18.8% [0.2%, 51.3%]	92.790%
	BMSCs	3	29.8% [12.3%, 50.6%]	52.086%
	UCMSCs	1	1.6% [0.6%, 3.4%]	/
Lower limb muscle atrophy	Total	1	16.7% [0.4%, 64.1%]	/
	BMSCs	1	16.7% [0.4%, 64.1%]	/
Osteoporosis	Total	1	15.0% [3.2%, 37.9%]	/
	UCMSCs	1	15.0% [3.2%, 37.9%]	/
Back pain	Total	1	4.5% [0.1%, 22.8%]	/
	UCMSCs	1	4.5% [0.1%, 22.8%]	/
Seizures	Total	1	3.4% [0.1%, 17.8%]	/
	NSCs	1	3.4% [0.1%, 17.8%]	/
**AEs of the digestive system**				
Vomit	Total	3	21.6% [8.9%, 37.2%]	0.0%
	BMSCs	1	23.1% [5.0%, 53.8%]	/
	ADMSCs	1	21.4% [4.7%, 50.8%]	/
	UCMSCs	1	20.0% [2.5%, 55.6%]	/
Gastrointestinal dysfunction	Total	4	10.8% [0.1%, 31.5%]	90.061%
	BMSCs	2	14.3% [5.7%, 25.6%]	0.0%
	UCMSCs	1	30.0% [11.9%, 54.3%]	/
	ESCs	1	1.0% [0.1%, 3.5%]	/
Nausea	Total	2	0.5% [0%, 2.5%]	71.1%
	ADMSCs	1	21.4% [4.7%, 50.8%]	/
	ESCs	1	1.0% [0.1%, 3.5%]	/
**AEs of the cardiovascular system**				
Hypertension	Total	2	12.7% [8.7%, 17.3%]	61.1%
	BMSCs	1	7.0% [1.5%, 19.1%]	/
	HSCs	1	14.4% [9.8%, 20.0%]	/
Low blood pressure	Total	1	5.9% [3.1%, 10.1%]	/
	HSCs	1	5.9% [3.1%, 10.1%]	/
Deep vein thrombosis	Total	1	5.0% [0.1%, 24.9%]	/
	UCMSCs	1	5.0% [0.1%, 24.9%]	/
Postoperative sepsis	Total	1	3.4% [0.1%, 17.8%]	/
	NSCs	1	3.4% [0.1%, 17.8%]	/
**Other AEs**				
Urinary tract infection	Total	3	23.7% [13.5%, 35.5%]	0.0%
	ADMSCs	1	21.4% [4.7%, 50.8%]	/
	UCMSCs	1	25.0% [8.7%, 49.1%]	/
	NSCs	1	24.1% [10.3%, 43.5%]	/
Lung infection	Total	1	20.0% [5.7%, 43.7%]	/
	UCMSCs	1	20.0% [5.7%, 43.7%]	/
Pressure ulcer	Total	2	14.1% [5.2%, 25.8%]	0.0%
	UCMSCs	1	10.0% [1.2%, 31.7%]	/
	NSCs	1	17.2% [5.8%, 35.8%]	/
Itching and rash	Total	2	1.6% [0.2%, 3.9%]	80.0%
	BMSCs	1	14.3% [4.8%, 30.3]	/
	ESCs	1	1.0% [0.1%, 3.5%]	/

The analysis of AEs based on the source of stem cells revealed that UCMSCs were associated with the most types of AEs (15 types), followed by BMSCs (12 types), NSCs (10 types), HSCs (9 types), ESCs (5 types), ADMSCs (4 types), and CBSCs (1 type). Further details are provided in Table 1.

#### Secondary outcome measures

**Fig. 3:**
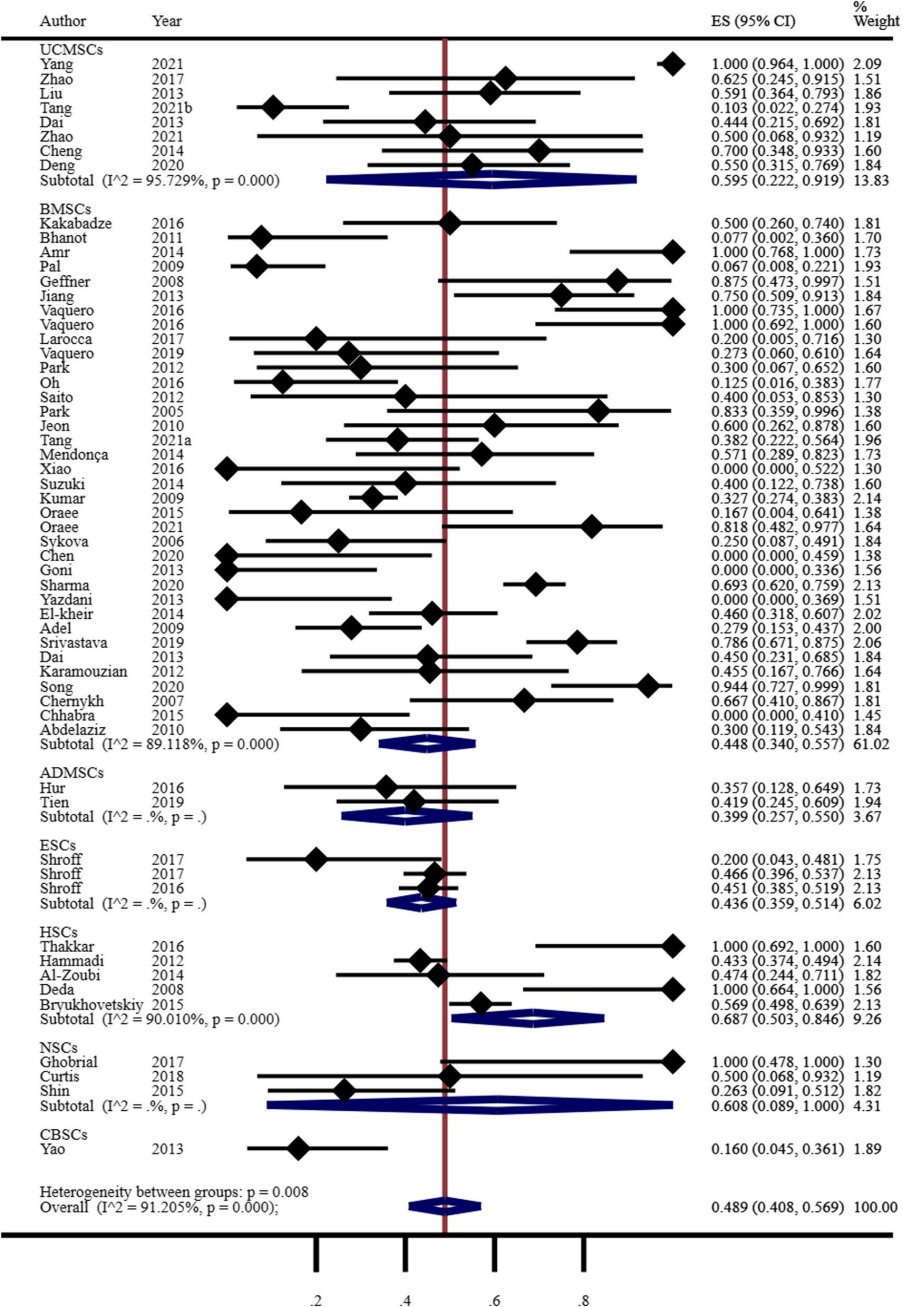
Findings from the meta-analysis of ASIA scores

The following are the secondary outcome measures:

1. A total of 58 studies reported improvements in ASIA scores among patients. The random effects model-based meta-analysis showed that stem cells improved motor function in 48.9% [40.8%, 56.9%] of patients with SCI. Through further subgroup analysis, the therapeutic effects of different types of stem cells were examined. HSCs were found to be the most effective, followed by NSCs, UCMSCs, BMSCs, ESCs, ADMSCs, and CBSCs, as shown in Fig. 3.
2. A total of 20 studies reported urinary system function improvements. The random effects model-based meta-analysis showed that stem cells improved urinary system function in 42.1% [27.6%, 57.2%] of patients with SCI. Further details are provided in Additional file 1: Fig. S1.
3. A total of 4 studies reported gastrointestinal function improvements. The random effects model-based meta-analysis showed that stem cells improved gastrointestinal function in 52.0% [23.6%, 79.8%] of patients with SCI. Further details are provided in Additional file 1: Fig. S2.

#### Publication bias and heterogeneity of included studies

**Fig. 4:**
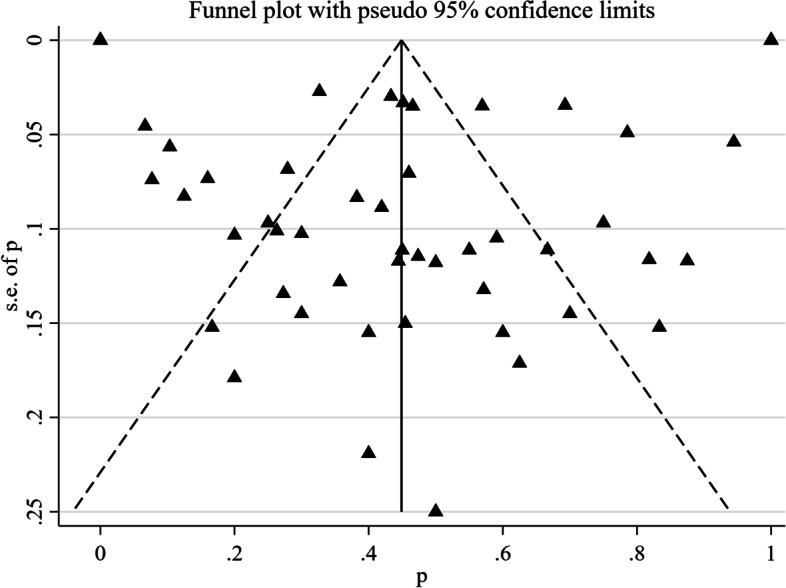
Funnel chart of ASIA scores

The publication bias test of ASIA shows that the funnel chart is basically symmetrical, suggesting that the possibility of publication bias is small, as shown in Fig. 4.

After quantitative assessment of heterogeneity among included studies by *I*^2^, we found that *I*^2^ > 50%, suggesting that there is heterogeneity among included studies. Even when we performed subgroup analyses of stem cells from different sources, heterogeneity persisted. This suggests another source of heterogeneity among studies. Through a retrospective analysis of the included studies, we found that the source of stem cells, transplantation dose, transplantation route, and transplantation timing varied widely among studies, as we mentioned in the basic information of the included studies. In addition, there were differences in age, degree of injury, injury time, and follow-up time of patients. All these factors may be sources of heterogeneity. The large difference prevented us from the further subgroup analysis. Because the subgroups are subdivided again, the number of studies within each subgroup will be small, or even lack of studies. Therefore, we have clarified the source of heterogeneity as much as possible, and hope that future research will be standardized according to relevant guidelines, to reduce the heterogeneity among different studies and improve the universality of the research results.

## Discussion

In recent years, the number of clinical trials based on stem cells has increased tremendously. Globally, there are now thousands of registered trials claiming to use “stem cells” in experimental treatments [85]. Thus, it appears that stem cell therapy has a well-established and strong clinical value. However, in reality, although some progress has been made, the clinical application of stem cells is still in its early stage. At present, clinical research on stem cells mainly includes phase I clinical trials, case series, and case reports. High-quality randomized controlled trials are lacking, and even simple controlled trials are few in number. Therefore, it is difficult to assess the efficacy of stem cells via head-to-head comparisons through meta-analysis [86]. In addition, although differences in cell types, sources, culture conditions, patient age, the degree of SCI, and other factors can make comparisons between studies challenging, these comparisons are still necessary [87]. Therefore, we analyzed the current evidence through a single-arm meta-analysis. Our findings showed that 48.9% of patients could benefit from stem cell therapy, i.e., 48.9% of patients showed at least one grade of improvement in the ASIA score after stem cell therapy. However, this only represents a slight improvement in sensory and motor function, far from the expected requirements for walking or daily activities. It is worth noting that the ASIA score-based assessment of sensory and motor function relies on subjective evaluation by the assessor and the patient, reducing result reliability to a certain extent [88]. Although this observed rate of 48.9% is lower than the rate of 78.57% obtained by Srivastava et al. in a randomized controlled trial with 200 patients [69] and the rate of 70% observed by Cheng et al. [31], given that small functional improvements are also extremely important for patient survival and well-being, stem cell therapy for SCI remains promising. Meanwhile, an increasing number of patients believe that the recovery of intestinal and bladder functions is as or more important than walking. Although our results show that the intestinal and bladder function improves in nearly half of all patients, these benefits only represent slight improvements in bladder fullness, urination impulse, and defecation (without sphincter control), which do not quite meet patient expectations [83].

Indeed, it is difficult to recruit enough SCI patients for clinical trials due to factors such as injury severity and patient age and physical condition. For cell-based trials, patients with SCI and grade A ASIA scores are recruited to avoid further damage. However, recovery in these patients may be low, and estimates of stem cell effectiveness may be inaccurate due to imprecisions in the measurement of subjective outcomes. Moreover, ASIA scale scores cannot fully reflect the severity of the pathology, and patients with the same ASIA grade may show a wide range of injury severity. Although the 48.8% rate of effectiveness appears encouraging, studies have shown that up to 70% of patients with complete cervical SCI can recover at least one spinal cord level within 1 year after injury [89]. Furthermore, 33% of patients with thoracic SCI have been found to show improvements of at least one grade [90]. Such spontaneous recovery in cases of SCI makes it difficult to confirm the efficacy of stem cells. Given that most trials lack a control group, it is not possible to rule out the contribution of spinal cord decompression or natural recovery to the therapeutic improvements observed after stem cell transplantation. Hence, stem cells cannot solely be credited with the therapeutic effects [90, 91]. Therefore, the actual therapeutic effects of the stem cells need to be further explored in standardized controlled trials according to the relevant guidelines.

While the extent to which stem cell therapy can benefit patients is unclear, the poor design and implementation of clinical trials also hinder the clinical applications of stem cells. Randomized controlled and double-blind human trials including placebo groups provide the most accurate and reliable data and are superior to observational studies or case reports [92]. However, most of the current studies are observational studies, case series, and so on. Small sample size and poor quality are also common problems in clinical trials [93]. Although the International Campaign for Cures of Spinal Cord Injury Paralysis (ICCP) has established a series of guidelines and standards for the design of clinical trials of SCI [92], 54.84% of the studies we examined were not registered on the clinical trial platform in advance. Hence, it was impossible to determine whether they reported all the results in accordance with the protocols and without any bias. The selective reporting of research results can lead to publication bias, thereby affecting the reliability of the conclusions garnered from a systematic review, or even leading to the opposite conclusion in some cases. Moreover, blinding is important to ensure the authenticity of clinical trial results. However, only 27.42% of studies included used blinding protocols. Failure to perform blinded evaluations may lead to the exaggeration of the actual effect and false-positive results [94]. Most of the included studies were phase I clinical trials, which are typically aimed at assessing the safety of stem cells. However, all studies explored and reported the efficacy of stem cells as the primary outcome measure, and ignored the reporting of AEs. Therefore, the safety of stem cells could be overestimated. At the same time, 35.48% of the studies followed patients for less than 1 year, which may have been insufficient to detect some AEs, such as tumor formation and inappropriate migration. Therefore, future clinical studies should extend the follow-up period as much as possible to fully explore the safety of stem cells. In addition, 79.03% of the studies did not pre-estimate the required number of patients to be enrolled in the trial or did not statistically analyze their results, reducing the likelihood of meaningful results. Most clinical trials do not have accurate and consistent inclusion and exclusion criteria for patients, leading to large differences in baseline characteristics, such as the location, type, injury duration, and severity of SCI. Moreover, the sources of stem cells and transplantation doses, timing, and routes also vary. This leads to study heterogeneity in meta-analyses and reduces the value of their findings in guiding future clinical trials. Hence, there is an urgent need to establish more stringent testing standards for stem cell therapy in SCI in order to standardize the design of clinical trials.

Patient safety should always be the top priority. The safety and AEs of stem cell therapy are mainly related to the inherent characteristics of the transplanted stem cells and the transplantation procedure. No serious AEs such as tumor formation were found in any of the studies we reviewed. Accordingly, these studies claimed that stem cell therapy was safe. However, it must be noted that the absence of serious AEs does not guarantee the safety of stem cell therapy. The 62 included studies reported a total of 28 AEs, including those affecting the nervous system, musculoskeletal system, digestive system, and cardiovascular system. Most of these AEs were mild, and the patients recovered completely after medical interventions. However, it is currently too soon to assume that stem cell therapy is safe, because this could further motivate those with blind confidence in stem cell therapy, which is not conducive to the scientific evaluation of its efficacy and safety. In addition, a study by Aspinall et al. showed that only 30% of clinical studies adequately reported various AEs during the clinical trial [95]. Hence, most studies may have ignored or even covered up AEs during the trial to “improve” the reported safety of stem cells. The meta-analysis by Zhao et al., which was based on control experiments, found a higher incidence of neuropathic pain and fever after stem cell therapy [96]. This was consistent with our findings, which indicated that the incidence of neuropathic pain, abnormal feeling, muscle spasms, vomiting, and urinary tract infection is higher than 20% in patients with SCI undergoing stem cell therapy. However, we reported a higher incidence of AEs than Zhao et al. because we enrolled more clinical trials, including single-arm studies. As a result, our sources of evidence were more comprehensive, and the results are more realistic. Although the observed AEs may not be related to the characteristics of the stem cells themselves, they could be affected by the transplantation technique, the severity of SCI, and the patients’ physical conditions. For example, neuropathic pain is mainly related to the use of large-caliber spinal needles during transplantation [97]. However, the observation of multiple AEs has greatly hindered the clinical applications of stem cells. In addition, after the subgroup analysis of stem cells from different sources, we found that mesenchymal stem cells such as BMSCs and UCMSCs—which were previously considered to have high safety—were associated with the highest rate of AEs among all the stem cells included in the current analysis [98]. This could be because the number of studies on BMSCs is the largest (61.29%, 38/62), and these studies also tend to have a large sample size. Hence, the AEs related to BMSCs are more apparent. In contrast, there was only one study on CBSCs. This study included 25 patients who were followed up for only 8 months. Hence, the AEs of CBSCs may be under-recognized. The possible AEs caused by stem cells can be explored properly only by reasonably calculating the required sample size, including patients who were clearly diagnosed with SCI, and ensuring a sufficient follow-up period. Hence, future studies should strongly focus on improving and standardizing these aspects.

Among the many safety issues surrounding stem cell transplantation, tumorigenesis is much more worrying than the fever and neuropathic pain caused by mild immune or allergic reactions [56, 79]. Stem cell products derived from hESCs and iPSCs confer the highest risk of tumorigenesis due to the presence of residual undifferentiated stem cells, malignantly transformed cells/mutations, and genetic instability. In addition, oncogenic activation due to the expression of foreign genes (e.g., various growth factors) and insertional mutagenesis of genetically modified viral vectors (e.g., retroviruses and lentiviruses) increases the risk of tumorigenicity and oncogenicity in stem cells [99]. For example, after iPSCs are treated with retroviruses, the retroviruses can get reactivated, resulting in tumor formation [100]. Although the tumorigenic potential of adult stem cells is thought to be lower than that of iPSC and hESCs, there have been reports of brain tumor formation after the injection of fetal NSCs [101]. Therefore, 71–74% of patients are “definitely” or “probably” not interested in participating in stem cell trials, and the possible risk of cancer is the greatest deterrent for these patients [46].

Currently, there is no global consensus on risk assessment strategies for the tumorigenicity and oncogenicity of stem cells. According to FDA guidelines, the risk of tumorigenesis can be decreased mainly by controlling the level of undifferentiated ESCs or other cellular impurities in hESC-derived cell products. The FDA guidelines also focus on the selection of preclinical animal species and the predictive ability of animal models to assess whether implanted cells form tumors at the transplant site and examine the malignant transformation of host cells and implanted cells in various tissues and organs. In fact, factors such as genetic and epigenetic variations in cell culture can increase the risk of tumorigenesis. Therefore, strategies to reduce the risk of tumors should be adopted during the development of high-risk stem cell products. Corresponding tumorigenicity testing methods can be established based on the quality and properties of candidate stem cells, the expected patient population, and tumorigenicity risk assessments. In preclinical studies, nude mice are usually used to study tumorigenicity in vivo, residual undifferentiated cells and transformed cells are used to study tumorigenicity in vitro, and server combined immune deficiency (SCID) mice are used to study tumorigenicity over the long term. No serious AEs like tumorigenesis have been reported in clinical trials so far, which could be due to the short follow-up period, because most of the studies (79%, 49/62) followed up patients for less than 2 years. Furthermore, the early termination of a trial is unfavorable for observing serious AEs such as tumor formation, especially because post-transplant ectopic growth can take up to 8 years [102, 103].

Although the success of stem cell therapy in preclinical studies has laid a good foundation for clinical research, its clinical translation has not been smooth. The number of new phase I and II clinical trials continued to increase from 2006 to 2012 but has since stagnated and even declined in 2018. The main reason is that the efficacy of stem cells is far less than expected [104]. First, animal studies attempt to minimize the variables in the experiment (e.g., baseline characteristics of the animals and the extent and location of lesions). However, patients with SCI show great heterogeneity (e.g., severity and location of injury, complications, age, sex, and rehabilitation training). Hence, the treatment efficacy observed in patients is often much lower than that observed in animals. Second, animal experiments often use the thoracic SCI model to study hindlimb motor recovery and related neural circuit changes. However, patients usually become quadriplegic due to cervical spine injury. In translational research, the extent and location of the lesion strongly influence the efficacy of the treatment. Some treatments yield beneficial effects only in subjects with specific lesion types [105]. Third, there are great differences in the inclusion and exclusion criteria among clinically recruited patients, and the location, severity, and timing of injury also differ. Therefore, it is difficult to include a homogenous group of patients even in high-quality randomized controlled trials, which makes the interpretation of treatment efficacy complicated and inaccurate. Fourth, the therapeutic effects observed in animal models are usually statistically significant. In clinical trials, the evaluation of functional results is more difficult because the small functional improvements generated by stem cells at the anatomical/histological level are difficult to detect. Finally, a careful investigation of the time window, dose, and route of stem cell transplantation is not a routine practice in animal studies, although these are major clinical problems that must be addressed in human studies [105].

In conclusion, the progress in clinical trials for stem cells has been exciting. However, most studies are in the early phase I/II stage, and clinical data are still being collected. It is too early to confirm that stem cells exert enormous curative effects. There are many differences and ambiguities in the selection of patients, cell types, timing of intervention, and doses and routes of stem cell transplantation in different clinical trials [75]. Therefore, close cooperation between preclinical and clinical aspects is warranted. Improving the safety, efficacy, and reproducibility of trials; identifying optimal transplantation parameters; strictly evaluating the benefits and risks of stem cell therapy; and strengthening supervision methods are urgent needs in this field [106, 107].

## Strengths and limitations

We estimated the efficacy of stem cell therapy and examined the rate of associated AEs to objectively and comprehensively analyze the feasibility of clinical translation, which is key for guiding future research. To our knowledge, this study is the first to perform such an analysis.

Nevertheless, there are some undeniable limitations to this study. First, there were great differences in the baseline characteristics of patients, types of stem cells, and timing, dose, and route of transplantation among the studies. Hence, we were unable to conduct further subgroup analysis and obtain more information. Second, real stem cells show self-renewal and can differentiate into various types of cells. In practical clinical research, the definition of stem cells is not strict. Bone marrow mononuclear cells and neural progenitor cells are also sometimes used as stem cells [9, 108]. In the absence of a clear definition, we included studies that claimed to examine “stem cells,” but some of these studies may have actually used stem cell-like cells. Third, we included only studies in English and did not examine grey literature and conference abstracts. As a result, there may have been some language bias, and some important findings may have been left out. Finally, although we developed a protocol and conducted the study in strict accordance with this protocol, we did not register it in PROSPERO or other comparable databases in advance, which may have led to reporting bias.

## Similarities and differences with similar studies

In order to better highlight the purpose and value of this study, we compared it with previously published studies. A total of 8 systematic reviews/meta-analyses (SR/MA) of stem cells for SCI were searched [20, 21, 109–114], as detailed in Table 2. Although safety is paramount for the clinical application of any therapy, we found that the published SR/MA focused on efficacy indicators while largely ignoring the safety outcomes of stem cell therapy. Although 7 of the 8 studies reported AEs, their reporting was not comprehensive. The AEs reported in these studies mainly included fever, headache, and neuropathic pain, but our study reported 28 kinds of AEs caused by stem cell transplantation, of which neuropathic pain, abnormal feeling, muscle spasms, vomiting, and urinary tract infections showed an incidence of > 20%. We calculated the incidence of various AEs and highlighted the common types of AEs. In addition, the types of studies we included were more extensive, the number of studies was larger, and the data extracted were richer. Therefore, our study can provide abundant data on the safety of stem cells and can guide future research.

**Table 2:** Similarities and differences with similar studies

Reference	Analyze methods	Number of studies	Sample size	Safety assessment (number of AEs)	Effectiveness assessment	Main conclusions
Our study	Meta-analysis of single-arm	62	2439	28	ASIA impairment scale grade, urinary system function, gastrointestinal system function	1) Stem cells can improve ASIA grade, urinary system function, and gastrointestinal system function. 2) Stem cell transplantation may cause 28 kinds of AEs, of which the incidence of neuropathic pain, abnormal feeling, muscle spasms, vomit, and urinary tract infection exceeds 20%.
Li et al. [111]	Meta-analysis of head-to-head comparisons	7	275	5	ASIA impairment scale grade, residual urine volume	1) Compared with conventional treatment, stem cells can significantly improve ASIA and improve residual urine volume to a certain extent. 2) More AEs occurred after stem cell transplantation, mainly including fever (22.2%), headache and dizziness (13.1%), neuropathic pain (10.6%), myologic pain (5.09%), and skin rash and itching (3.32%).
Fan et al. [113]	Meta-analysis of head-to-head comparisons	10	377	5	ASIA impairment scale grade, ADL score, residual urine volume	1) Compared with rehabilitation therapy, stem cells can significantly improve ASIA grade and residual urine volume, but have no significant effect on ADL score. 2) Stem cell transplantation has a higher incidence of AEs (RR = 14.49, 95%CI [5.34, 34.08], *p* < 0.00001), mainly including fever, headache, backache, numbness, and abdominal distension.
Xu et al. [109]	Meta-analysis of head-to-head comparisons	11	499	5	ASIA impairment scale grade, ADL score, residual urine volume	1) Compared with conventional treatment, stem cells can significantly improve ASIA, ADL, and residual urine volume. 2) There were more AEs after stem cell transplantation (RR = 20.34, 95%CI [8.09–51.18], *p* < 0.001), mainly including fever, headache, backache, numbness, and abdominal distension.
Muthu et al. [110]	Meta-analysis of head-to-head comparisons	19	670	3	ASIA impairment scale grade, ADL score, residual urine volume, bladder function improvement, SSEP improvement	1) The stem cells group showed statistically significant improvement in ASIA grade, bladder function, residual urine volume, and SSEP. However, no significant difference was noted in ADL. 2) More AEs occurred after stem cell transplantation (RR = 4.342, 95%CI [2.248, 6.436], *p* < 0.001), mainly including fever, headache, and neuropathic pain.
Liu et al. [21]	Network meta-analysis	12	642	3	ASIA impairment scale grade, Barthel index	1) Stem cells combined with rehabilitation training were significantly more effective than rehabilitation training alone in improving ASIA and Barthel index. 2) More adverse effects occurred after stem cell transplantation, mainly including fever (7.59%), headache (5.69%), and neuropathic pain (4.88%).
Tang et al. [20]	Meta-analysis of head-to-head comparisons	9	328	3	ASIA impairment scale grade; urodynamic indices	1) Compared with conventional treatment, stem cells can significantly improve ASIA and improve bladder function. 2) More AEs occurred after stem cell transplantation, mainly including neuropathic pain (RR = 1.58, 95%CI [0.92, 2.72], *p* = 0.10), fever (RR = 4.22, 95%CI [1.74, 10.22], *p* = 0.001), and headache (RR = 2.40, 95%CI [0.57, 10.17], *p* = 0.23).
Johnson et al. [112]	Meta-analysis of head-to-head comparisons	19	224	0	ASIA impairment scale grade	Compared with conventional treatment, stem cells can significantly improve ASIA.
Chen et al. [114]	Network meta-analysis	18	949	4	ASIA impairment scale grade, Barthel index	1) Stem cells significantly improved ASIA grade and Barthel index compared to rehabilitation. 2) More AEs occurred after stem cell transplantation (OR = 14.35, 95%CI [4.28, 48.07], *p* < 0.001), mainly including fever, headache, back pain, and numbness.

*AEs* Adverse events, *ASIA* American Spinal Injury Association, *ADL* Activities of daily living, *SSEP* Somatosensory evoked potential

## Conclusions

To facilitate the translation of stem cell therapy from the bench to the bedside, stem cells must be fully proven to be safe and effective in animal and clinical studies. The effectiveness of stem cells is very clear in rodent models. However, our systematic review of clinical trials found that although stem cells have great potential in improving neurological function in patients with SCI, stem cell transplantation could cause 28 kinds of AEs. Some of these AEs may be potentially serious and cannot be ignored, even if they have not been detected. Current clinical trials have small samples, low quality, and lack control groups. Thus, they cannot demonstrate the safety of stem cell therapy completely. Therefore, the possible AEs caused by stem cell transplantation need to be properly explored in preclinical large animal models and early clinical studies. Furthermore, optimal transplantation conditions and parameters need to be identified to improve the therapeutic effects of stem cells. At the same time, experts in the field must pay attention to safety concerns, as the proven safety of stem cell therapy will be paramount for its clinical adoption. Our findings show that the current evidence is insufficient to advocate for the widespread use of stem cell therapy for SCI and warn against its rapid clinical translation. Until well-designed animal experiments and high-quality clinical studies are performed, the introduction of stem cell therapy into the clinic should be slow and performed with caution.

## Supplementary Information

**Additional file 1: Table S1.** Search strategies. **Table S2.** Basic information of the included studies. **Fig. S1.** Findings from the meta-analysis of improvement in urinary system. **Fig. S2.** Findings from the meta-analysis of improvement in gastrointestinal function.

## Data Availability

The datasets used and/or analyzed during the current study are available from the corresponding author on reasonable request.

## Abbreviations

ADL: Activities of daily living
ADMSCs: Adipose tissue-derived mesenchymal stem cells
AEs: Adverse events
ASIA: American Spinal Cord Injury Association Impairment Scale
BMSCs: Bone marrow mesenchymal stem cells
CBSCs: Cord blood stem cells
ESCs: Embryonic stem cells
FDA: Food and Drug Administration
hESCs: Human embryonic stem cells
HSCs: Hematopoietic stem cells
ICCP: International Campaign for Cures of Spinal Cord Injury Paralysis
iPSCs: Induced pluripotent stem cells
MINORS: Methodological index for non-randomized study
NSCs: Neural stem cells
SCI: Spinal cord injury
SCID: Server combined immune-deficiency
SR/MA: Systematic review/meta-analysis
SSEP: Somatosensory evoked potential
UCMSCs: Umbilical cord mesenchymal stem cells
