# Supplementary material for: Clinical translation of stem cell therapy for spinal cord injury still premature: results from a single-arm meta-analysis based on 62 clinical trials
Source: BMC Med. 2022 Sep 5;20:284. doi: 10.1186/s12916-022-02482-2 (PMC9442938; doi:10.1186/s12916-022-02482-2)
Supplement: Supplementary file 1 — Additional file 1: Table S1. Search strategies. Table S2. Basic information of the included studies. Fig. S1. Findings from the meta-analysis of improvement in urinary system. Fig. S2. Findings from the meta-analysis of improvement in gastrointestinal function. [file 12916_2022_2482_MOESM1_ESM.docx]

**Additional file 1**

**Table S1:** **Search strategies**

| PubMed  #1: "Spinal Cord Injuries"[Mesh] 52,590  #2: "Spinal cord injury"[Title/Abstract] OR "Spinal injury"[Title/Abstract] OR "Spinal Cord Trauma"[Title/Abstract] OR "Spinal Cord Transection"[Title/Abstract] OR "Spinal Cord Laceration"[Title/Abstract] OR "Post-Traumatic Myelopathy"[Title/Abstract] OR "Spinal Cord Contusion"[Title/Abstract] 42,994  #3: #1 OR #2 66,574  #4: "Stem Cells"[Mesh] 242,774  #5: "stem cell"[Title/Abstract] OR "stem cells"[Title/Abstract] 306,870  #6: #4 OR #5 398,140  #7 clinical [Title/Abstract] OR trials [Title/Abstract] OR trial [Title/Abstract] OR patients [Title/Abstract] OR patient [Title/Abstract] 9,475,647  #8: #3 AND #6 AND #7 1028  Web of science  ((TS= (“Spinal cord injury” OR “Spinal injury” OR “Spinal Cord Trauma” OR “Spinal Cord Transection” OR “Spinal Cord Laceration” OR “Post-Traumatic Myelopathy” OR “Spinal Cord Contusion”)) AND TS= (“stem cell” OR “stem cells”)) AND TS= (clinical OR trials OR trial OR patients OR patient) 3630  Embase  #1: (Spinal cord injury or Spinal injury or Spinal Cord Trauma or Spinal Cord Transection or Spinal Cord Laceration or Post-Traumatic Myelopathy or Spinal Cord Contusion).mp. [mp=title, abstract, heading word, drug trade name, original title, device manufacturer, drug manufacturer, device trade name, keyword heading word, floating subheading word, candidate term word] 70684  #2: *spinal cord injury/ 36035  #3: *spinal cord transection/ 933  #4: 1 or 2 or 3 70900  #5: (stem cell or stem cells).mp. [mp=title, abstract, heading word, drug trade name, original title, device manufacturer, drug manufacturer, device trade name, keyword heading word, floating subheading word, candidate term word] 623515  #6: exp stem cell/ 411768  #7: 5 or 6 645441  #8: (clinical or trials or trial or patients or patient).mp. [mp=title, abstract, heading word, drug trade name, original title, device manufacturer, drug manufacturer, device trade name, keyword heading word, floating subheading word, candidate term word] 15047484  #9: exp clinical trial/ 1635728  #10: *patient/ 376328  #11: exp clinical study/ or exp clinical research/ 9882269  #12: 8 or 9 or 10 or 11 15998259  #13: 4 and 7 and 12 2239  Cochrane  #1: MeSH descriptor: [Spinal Injuries] explode all trees 865  #2 MeSH descriptor: [Spinal Cord Injuries] explode all trees 1857  #3 (Spinal cord injury OR Spinal injury OR Spinal Cord Trauma OR Spinal Cord Transection OR Spinal Cord Laceration OR Post-Traumatic Myelopathy OR Spinal Cord Contusion): ti,ab,kw 5177  #4 #1 or #2 or #3 5873  #5 MeSH descriptor: [Stem Cells] explode all trees 887  #6 (stem cell OR stem cells):ti,ab,kw 14257  #7 #5 or #6 14356  #8 #4 and #7 73 |
| --- |

**Table S2:** **Basic information of the included studies**

| **NO.** | **Author** | **Year** | **Country** | **Type of studies** | **Sample size** | **Age** | **Gender (M/F)** | **Spinal cord injury** | | **Stem cell** | | | | **Follow-up time (months)** |
| --- | --- | --- | --- | --- | --- | --- | --- | --- | --- | --- | --- | --- | --- | --- |
|  |  |  |  |  |  |  |  | **Injury site** | **Damage time/months** | **Type** | **Source** | **Dose** | **Transplant route** |  |
| 1 | Abdelaziz [23] | 2010 | Egypt | Control | 20 | 6-64 | 19/1 | / | / | BMSCs | Autologous iliac bone marrow | 5×10^6^/kg | Intrathecal+Intralesional | 12 |
| 2 | Adel [24] | 2009 | Egypt | Control | 43 | 31.7±10.4 | 36/7 | Cervical and thoracic spine | 43.2 | BMSCs | Autologous iliac bone marrow | 5-10×10^6^ | Intrathecal | 6 |
| 3 | Albu [25] | 2021 | Spain | Control | 10 | 25-47 | 7/3 | Thoracic spine | / | UCMSCs | Allogeneic neonatal umbilical cord tissue | 1×10^7^ | Intrathecal | 12 |
| 4 | Al-Zoubi [26] | 2014 | USA | Single-arm | 19 | 20-49 | 16/3 | Thoracic spine | 12-48 | HSCs | 自体外周血 | 7.6×10^7^ | Intrathecal | 60 |
| 5 | Amr [27] | 2014 | Egypt | Single-arm | 14 | 9-45(22) | 12/2 | Thoracic spine | 5-84 | BMSCs | Autologous iliac bone marrow | / | Scaffold | 24 |
| 6 | Bhanot [28] | 2011 | India | Single-arm | 13 | 18-60(32.2) | 10/5 | Cervical and thoracic spine(Cervical5，Thoracic8) | 3-132 | BMSCs | Autologous iliac bone marrow | 3-8×10^6^/kg | Intrathecal | 6-38 |
| 7 | Bryukhovetskiy [29] | 2015 | Russian | Control | 202 | 19-51 | 156/46 | Unlimited(Cervical98，Thoracic93，lumbar11) | / | HSCs | Autologous peripheral blood | 5.8×10^6^ | Intrathecal | 36-60 |
| 8 | Chen [30] | 2020 | China | Single-arm | 7 | 29-61(46.57) | 7/0 | Thoracic spine | 3-27 days | BMSCs | Autologous iliac bone marrow | >1×10^9^ | Scaffold | 36 |
| 9 | Cheng [31] | 2014 | China | Control | 10 | 35.30 ± 8.23 | / | Thoracolumbar spine | 21.40 ± 12.96 | UCMSCs | Allogeneic neonatal umbilical cord tissue | 4×10^7^ | Intralesional | 6 |
| 10 | Chernykh [32] | 2007 | Russian | Control | 18 | 18-47(31.6) | 14/4 | Unlimited | 36.4±7.9 | BMSCs | Autologous bone marrow | / | Intralesional+Intravenous | 9.4±4.6 |
| 11 | Chhabra [33] | 2015 | India | Control | 7 | 20-30 | 6/1 | Thoracic spine | / | BMSCs | Autologous iliac bone marrow | 2×10^8^ | Intrathecal | 12 |
| 12 | Curtis [34] | 2018 | USA | Single-arm | 4 | 25-35(29.75) | 3/1 | Thoracic spine | / | NSCs | / | / | Intralesional | 18-27 |
| 13 | Dai [35] | 2013 | China | Control | 18 | 36±9.68 | 16/2 | Cervical and thoracic spine | 18.67±7.68 | UCMSCs | Allogeneic neonatal umbilical cord tissue | 4×10^7^ | Intralesional | 6 |
| 14 | Dai [36] | 2013 | China | Control | 20 | 22-54(34.7±8.9) | 14/6 | Cervical spine | 51.9±18.3 | BMSCs | Autologous iliac bone marrow | 2×10^7^ | Intralesional | 6 |
| 15 | Deda [37] | 2008 | Turkey | Single-arm | 9 | 17-40 (28.11) | 5/4 | Cervical and thoracic spine(Cervical6，Thoracic3) | 24-204 | HSCs | Autologous iliac bone marrow | 5×10^6^ | Intrathecal | 24 |
| 16 | Deng [38] | 2020 | China | Control | 20 | 33.70±9.03 | 5/15 | Cervical spine | 12.45±5.74 | UCMSCs | Allogeneic neonatal umbilical cord tissue | 4×10^7^ | Scaffold | 12 |
| 17 | El-kheir [39] | 2014 | Egypt | Control | 50 | 16-45 | / | Cervical and thoracic spine(Cervical10，Thoracic40) | 12-36(18.25 ± 5) | BMSCs | Autologous iliac bone marrow | 2×10^6^/kg | Intrathecal | 18 |
| 18 | Geffner [40] | 2008 | Ecuador | Single-arm | 8 | 27-44 | 7/1 | Thoracic spine | 1-262 | BMSCs | Autologous iliac bone marrow | 1.2×10^6^/kg | Intravenous+spinal canal | 24 |
| 19 | Ghobrial [41] | 2017 | USA | Single-arm | 5 | 24-49(34.2) | 5/0 | Cervical spine | / | NSCs | Allogeneic fetus | 1.5-4×10^7^ | Intrathecal | 12 |
| 20 | Goni [42] | 2013 | India | Single-arm | 9 | 18-55(30.3) | 8/1 | Thoracic spine | >6 | BMSCs | Autologous iliac bone marrow | / | Intrathecal | 24 |
| 21 | Hammadi [43] | 2012 | Iraq | Single-arm | 277 | 18-65(34.5) | 252/25 | Cervical and thoracic spine(Cervical69，Thoracic208) | 6-104 | HSCs | Autologous peripheral blood | 1-8×10^8^ | Intrathecal | 24 |
| 22 | Hur [44] | 2016 | Korea | Single-arm | 14 | 20-66(13.7±7.6) | 12/2 | Unlimited(Cervical6，CervicalThoracic1，Thoracic6，lumbar1) | 3-28 | ADMSCs | Autologous subcutaneous fat | 9×10^7^ | Intrathecal | 8 |
| 23 | Jeon [45] | 2010 | Korea | Single-arm | 10 | 34-61(46) | 8/2 | Cervical spine | 1-108 | BMSCs | Autologous iliac bone marrow | 8×10^6^ | Intrathecal | 6 |
| 24 | Jiang [46] | 2013 | China | Single-arm | 20 | 9-72(41.1) | 13/7 | Unlimited(Cervical4，Thoracic11，lumbar5) | 3-120 | BMSCs | Autologous iliac bone marrow | 1×10^8^ | Intrathecal | 1 |
| 25 | Kakabadze [47] | 2016 | Georgia | Single-arm | 18 | 22-65 | 13/5 | Thoracolumbar spine(Thoracic12，lumbar6) | 5-20 | BMSCs | Autologous iliac bone marrow | 405-964×10^6^ | Intrathecal | 12 |
| 26 | Karamouzian [48] | 2012 | Iran | Control | 11 | 23-48(33.18±8.9) | 7/4 | Thoracic spine | 20.3 | BMSCs | Autologous iliac bone marrow | 7×105-1.2×10^6^ | Intrathecal | 12-33 |
| 27 | Kumar [49] | 2009 | India | Single-arm | 297 | / | / | / | / | BMSCs | Autologous iliac bone marrow | / | Intrathecal | 18.4-20.5 |
| 28 | Larocca [50] | 2017 | Brazil | Single-arm | 5 | 26-52(44.8) | 5/0 | Thoracic spine | >12 | BMSCs | Autologous iliac bone marrow | 2×10^7^ | Subcutaneous | 6 |
| 29 | Levi [51] | 2017 | USA | Single-arm | 29 | 18-49 | 27/2 | Cervical and thoracic spine(Cervical12，Thoracic17) | / | NSCs | / | 1.5-4×10^7^ | Intralesional | >12 |
| 30 | Levi [52] | 2018 | USA | Control | 12 | 22-28 | 11/1 | Cervical spine | 4-24 | NSCs | / | 15-40×10^6^ | Intralesional | 12 |
| 31 | Liu [53] | 2013 | China | Single-arm | 22 | 18-51(33) | 17/5 | Cervical and thoracic spine(Cervical4，CervicalThoracic2，Thoraciclumbar2，lumbar7) | 2-204 | UCMSCs | Allogeneic neonatal umbilical cord tissue | 4×10^6^/kg | Intrathecal | >12 |
| 32 | Mendonça [54] | 2014 | Brazil | Single-arm | 14 | 23-61 | 10/4 | Thoracolumbar spine | 18-180 | BMSCs | Autologous iliac bone marrow | 5×10^6^ /cm3 | Intralesional | 6 |
| 33 | Oh [55] | 2016 | Korea | Single-arm | 16 | 16-65(40.9) | 15/1 | Cervical spine | 24-181 | BMSCs | Autologous iliac bone marrow | 4.8×10^7^ | Subdural | 6 |
| 34 | Oraee [56] | 2015 | Iran | Single-arm | 6 | 22-45(33.3) | 4/2 | Cervical and thoracic spine(Cervical1，Thoracic5) | 38.1±15.28 | BMSCs | Autologous iliac bone marrow | 1×10^6^ | Intrathecal | 30 |
| 35 | Oraee [57] | 2021 | Iran | Single-arm | 11 | 17-42(32) | 8/2 | Cervical and thoracic spine(Cervical4，Thoracic6) | / | BMSCs | Autologous iliac bone marrow | 5×10^7^ | Intrathecal | 12 |
| 36 | Pal [58] | 2009 | India | Single-arm | 30 | / | 27/3 | Cervical and thoracic spine | >1 | BMSCs | Autologous iliac bone marrow | 1×10^6^/kg | Intrathecal | 12-36 |
| 37 | Park [59] | 2012 | Korea | Single-arm | 10 | 34-61 | / | Cervical spine | >1 | BMSCs | Autologous iliac bone marrow | 8×10^6^ | Intralesional+Subdural | 6 |
| 38 | Park [60] | 2005 | Korea | Single-arm | 6 | 17-51(35.83) | 6/0 | Cervical spine | / | BMSCs | Autologous iliac bone marrow | 1.98×10^10^ | Intralesional | 6-18 |
| 39 | Saito [61] | 2012 | Japan | Single-arm | 5 | 23-59(42.6) | / | Cervical spine | / | BMSCs | Autologous iliac bone marrow | 3-5×10^7^ | Intrathecal | 12-48 |
| 40 | Satti [62] | 2016 | Pakistan | Single-arm | 9 | 24-38(35.5) | / | Thoracic spine | 10-55 | BMSCs | Autologous iliac bone marrow | 1.2×10^6^/kg | Intrathecal | 12-24 |
| 41 | Sharma [63] | 2020 | India | Single-arm | 180 | 32.3 | 160/20 | Cervical and thoracic spine(Cervical63，Thoracic117) | / | BMSCs | Autologous iliac bone marrow | 1.06×10^8^ | Intrathecal | 9±7 |
| 42 | Shin [64] | 2015 | Korea | Control | 19 | 18-57(37.2) | 16/3 | Cervical spine | 7-168 days | NSCs | Human fetal brain | 1×10^8^ | Intralesional | 6 |
| 43 | Shroff [65] | 2017 | India | Single-arm | 15 | 15-44(27) | 13/2 | / | 10-467 days | ESCs | / | 1.6×10^7^ | Intravenous | 9 |
| 44 | Shroff [66] | 2017 | India | Single-arm | 226 | 20-34(28) | 167/59 | Unlimited | >3 | ESCs | / | 1.6×10^7^ | Intravenous | >12 |
| 45 | Shroff [67] | 2016 | India | Single-arm | 226 | 20-34(28) | 167/59 | Unlimited | >12 | ESCs | / | / | / | / |
| 46 | Song [68] | 2020 | China | Control | 18 | 19-59(41.2±2.3) | 12/6 | Unlimited | / | BMSCs | Autologous iliac bone marrow | 1×10^7^ | Intrathecal | 12 |
| 47 | Srivastava [69] | 2019 | India | Control | 70 | 30.84 ± 10.56 | 63/7 | / | / | BMSCs | Autologous iliac bone marrow | / | Intrathecal | 12 |
| 48 | Suzuki [70] | 2014 | Japan | Single-arm | 10 | 20-57(30.7) | 10/0 | Cervical and thoracic spine | / | BMSCs | Autologous iliac bone marrow | 2.03-8.44×10^8^ | Intrathecal | 6 |
| 49 | Sykova [71] | 2006 | Czech Republic | Single-arm | 20 | 19-41 | 16/4 | Thoracic spine | 10-467 days | BMSCs | Autologous iliac bone marrow | 104.0 ± 55.3 × 10^8^ | Intravenous+arterial | 24 |
| 50 | Tang [72] | 2021a | China | Single-arm | 34 | 22-51 | / | Cervical and thoracic spine | 2-80 | BMSCs | Autologous iliac bone marrow | 4×10^7^ | Scaffold | >12 |
| 51 | Tang [73] | 2021b | China | Single-arm | 29 | 22-51 | / | Cervical and thoracic spine | 2-80 | UCMSCs | Allogeneic neonatal umbilical cord tissue | 1×10^9^ | Scaffold | >24 |
| 52 | Thakkar [73] | 2016 | India | Single-arm | 10 | 9-42(28) | 8/2 | Thoracolumbar spine | 30-64.8 | HSCs | Autologous bone marrow + abdominal adipose tissue | 1.82×10^8^ | Intrathecal | 34 |
| 53 | Tien [74] | 2019 | Vietnam | Control | 31 | / | / | / | / | ADMSCs | Autologous adipose tissue | >1×10^8^ | Intrathecal | 12 |
| 54 | Vaquero [75] | 2016a | Spain | Single-arm | 12 | 40.5±8.75 | 9/3 | Thoracic spine | 38-321 | BMSCs | Autologous iliac bone marrow | 1-2.3×10^8^ | Intrathecal | 12 |
| 55 | Vaquero [76] | 2016b | Spain | Single-arm | 10 | 34-59(42.2±9.3) | 8/2 | Cervical and lumbar spine | 29-415 | BMSCs | Autologous iliac bone marrow | 3×10^7^ | Intrathecal | 12 |
| 56 | Vaquero [77] | 2018 | Spain | Single-arm | 11 | 28-62(44.91±10.17 ) | 7/4 | Unlimited(Cervical4，Thoracic4，lumbar3) | 163.8±177.48 | BMSCs | Autologous iliac bone marrow | 3×10^8^ | Intrathecal | 10 |
| 57 | Xiao [78] | 2016 | China | Single-arm | 5 | 27-56(43.2) | 4/1 | Cervical and thoracic spine(Cervical1，Thoracic4) | 2-32 | BMSCs | Autologous iliac bone marrow | 1×10^9^ | Scaffold | 12 |
| 58 | Yang [79] | 2021 | China | Single-arm | 102 | 18-65(38.1±11) | / | Unlimited | 2-240 | UCMSCs | Allogeneic neonatal umbilical cord tissue | 1×10^6^/kg | Intrathecal | 12 |
| 59 | Yao [80] | 2013 | China | Control | 25 | 18-48(36.7±3.8) | 16/9 | Unlimited(Cervical5，Thoracic11，lumbar9) | >6 | CBSCs | Allogeneic umbilical cord blood | 1-3×10^7^ | Intrathecal+Intravenous | 8 |
| 60 | Yazdani [81] | 2013 | India | Single-arm | 8 | 15-45(30.5±8.15) | 4/4 | Cervical and thoracic spine(Cervical1，Thoracic7) | 39.80-19.07 | BMSCs | Autologous iliac bone marrow | 1×10^6^ | Intralesional | 24 |
| 61 | Yoon [82] | 2007 | Brazil | Control | 35 | 18-60 | 29/6 | Cervical and thoracic spine | >14 days | BMSCs | Autologous iliac bone marrow | 1×10^8^ | Intralesional | 10.4 |
| 62 | Zhao [83] | 2017 | China | Single-arm | 8 | 24-41(31.5) | 7/1 | Cervical and thoracic spine(Cervical4，Thoracic4) | 2-36 | UCMSCs | Allogeneic neonatal umbilical cord tissue | 4×10^7^ | Scaffold | 12 |
| 63 | Zhao [84] | 2021 | China | Control | 7 | 22-47(32.43) | 6/1 | Cervical and thoracic spine(Cervical3，Thoracic4) | >1 | UCMSCs | Allogeneic neonatal umbilical cord tissue | 5×10^4^ | Intrathecal | 6 |

**
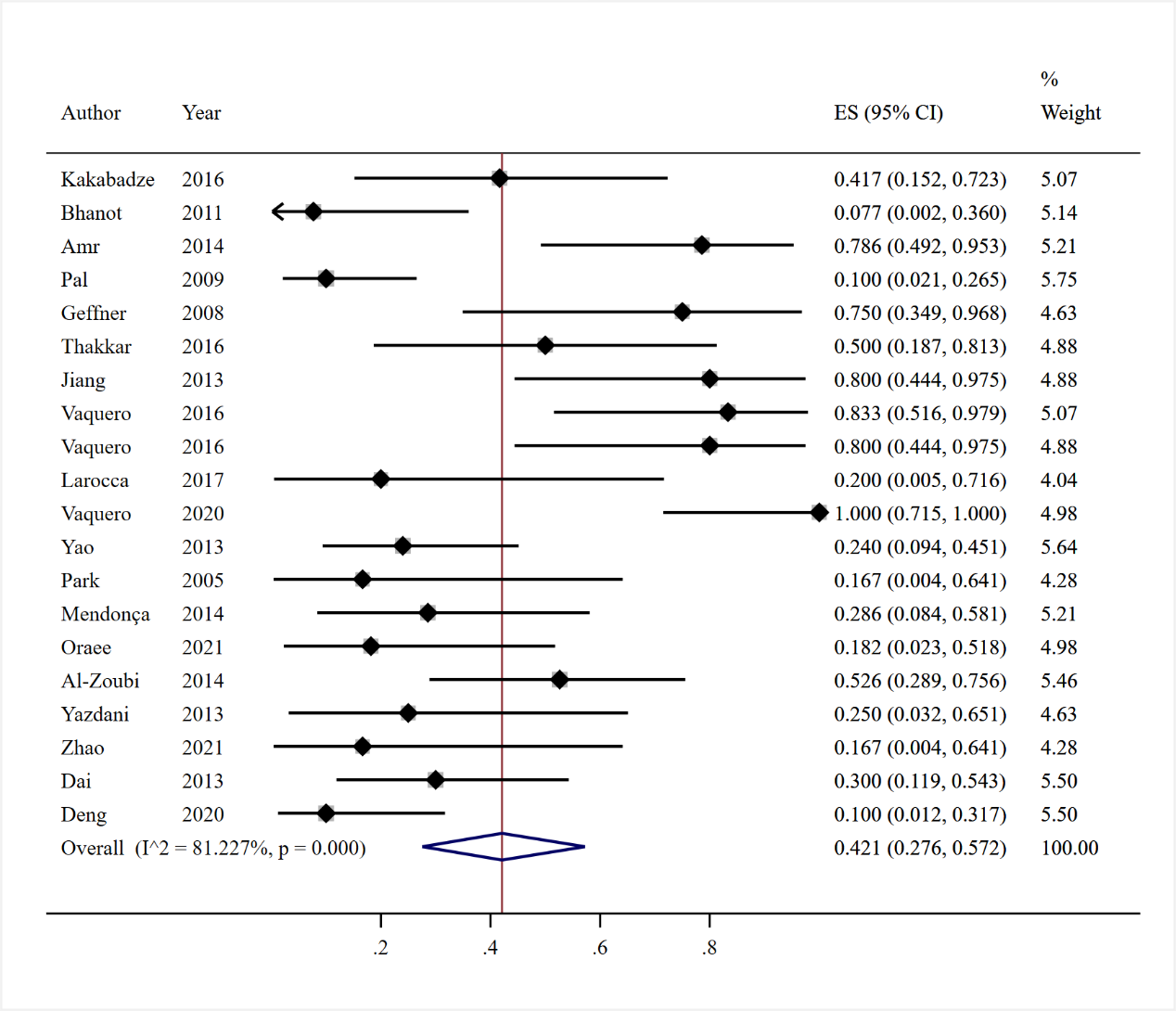
**

**Figure S1:** **Findings from the meta-analysis of improvement in urinary system.**

**
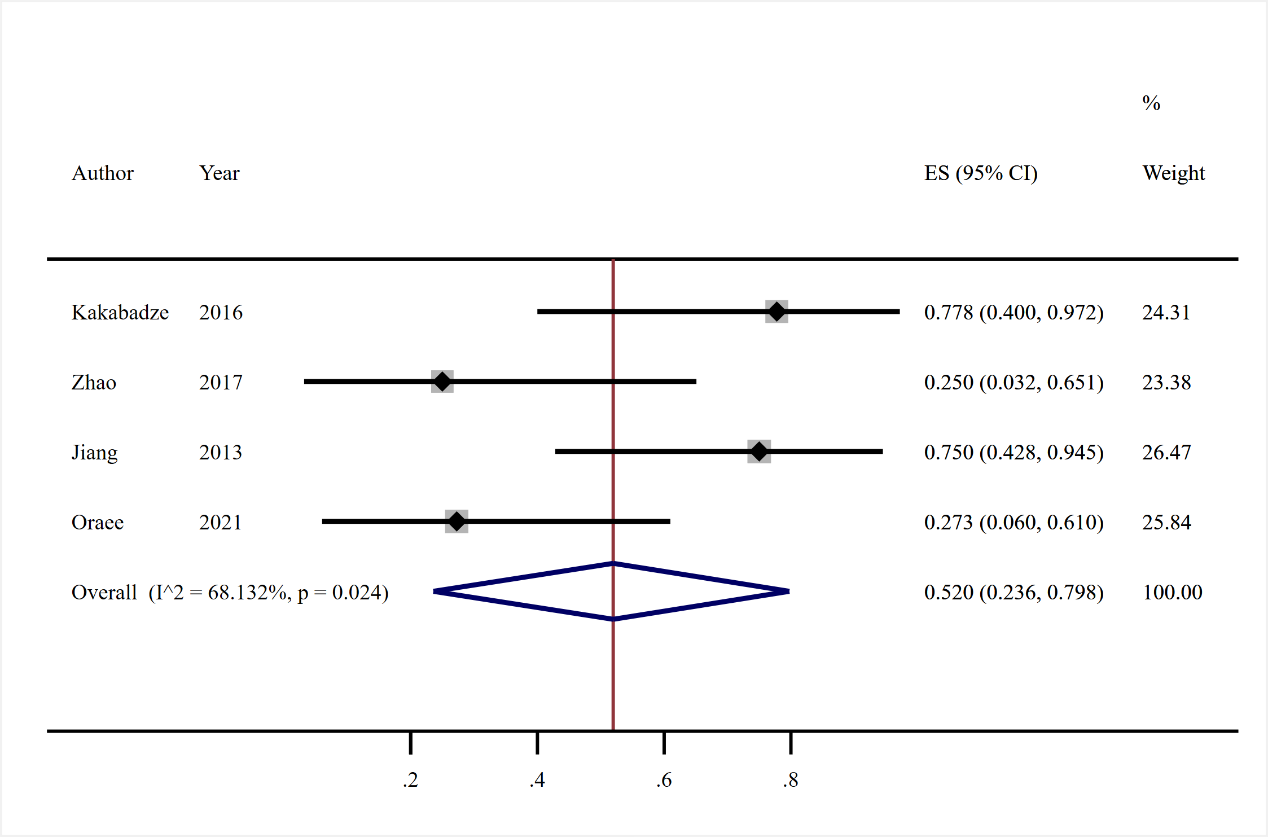
**

**Figure S2:** **Findings from the meta-analysis of improvement in gastrointestinal function.**
